# “Embodied Body Language”: an electrical neuroimaging study with emotional faces and bodies

**DOI:** 10.1038/s41598-017-07262-0

**Published:** 2017-07-31

**Authors:** Marta Calbi, Monica Angelini, Vittorio Gallese, Maria Alessandra Umiltà

**Affiliations:** 1 Department of Medicine and Surgery, Unit of Neuroscience, University of Parma, Parma, Italy; 2 Department of Clinical and Experimental Sciences, University of Brescia, Brescia, Italy; 3 Institute of Philosophy, School of Advanced Study, University of London, London, UK; 4 Department of Food and Drug Sciences, University of Parma, Parma, Italy

## Abstract

To date, most investigations in the field of affective neuroscience mainly focused on the processing of facial expressions, overlooking the exploration of emotional body language (EBL), its capability to express our emotions notwithstanding. Few electrophysiological studies investigated the time course and the neural correlates of EBL and the integration of face and body emotion-related information. The aim of the present study was to investigate both the time course and the neural correlates underlying the integration of affective information conveyed by faces and bodies. We analysed EEG activities evoked during an expression matching task, requiring the judgment of emotional congruence between sequentially presented pairs of stimuli belonging to the same category (face-face or body-body), and between stimuli belonging to different categories (face-body or body-face). We focused on N400 time window and results showed that incongruent stimuli elicited a modulation of the N400 in all comparisons except for body-face condition. This modulation was mainly detected in the Middle Temporal Gyrus and within regions related to the mirror mechanism. More specifically, while the perception of incongruent facial expressions activates somatosensory-related representations, incongruent emotional body postures also require the activation of motor and premotor representations, suggesting a strict link between emotion and action.

## Introduction

For a long time, the field of Affective Neuroscience has been dominated by studies of emotional facial expressions. Nonetheless, it is now well agreed that also “emotional body language” (EBL) plays a fundamental role during social interactions, given the relevance of body postures in expressing our emotional and mental states^1–3^.

Neuroimaging studies revealed that the visual processing of human body is mainly underpinned by the extrastriate body area (EBA), in lateral occipitotemporal cortex^4, 5^, and by the fusiform body area (FBA), in the posterior fusiform gyrus^5–7^. Beyond these, other regions engaged during the elaboration of EBL include both emotion-related brain areas and networks for action representation, suggesting a link between emotion and action^8^. The existence of the human putative mirror mechanism for action (MM), whose activity is modulated by both action execution and action observation, provided new insights on the role of motor-related areas in perception of EBL. The MM for action, exemplified by the activation of the inferior parietal lobule (IPL), the inferior frontal gyrus (IFG), the premotor cortex (PMc) and the superior temporal sulcus (STS)^9, 10^, could indeed provide the neurobiological basis for many emotional and social cognitive skills^11, 12^.

According to the hypothesized “two-system” model of emotion-behaviour connectivity^12^, a first network including subcortical structures, would support the rapid and automatic perception of EBL. A second system (involving lateral occipital cortex, STS, intraparietal lobule, fusiform gyrus, amygdala and PMc), would further estimate the behavioural expression of a given emotion, deciding for the best response to the stimulus. Moreover, these two systems are linked to a third network, the “body awareness system”, which involves the insula, somatosensory cortex, anterior cingulate cortex (ACC) and ventromedial prefrontal cortex.

Previous event related potentials (ERPs) studies showed that the structural encoding of human bodies and body parts, as for faces, is indexed by the N170, a negative component peaking between 140 and 230 ms after stimulus presentation at occipitotemporal sites^13–15^.

Later on, semantic information conveyed by bodies, and the elaboration of action’s meanings, intentions and rules of execution are indexed by modulations of the N400 component.

The N400, of particular interest for the present study, is a negative component peaking around 400 ms after the onset of a meaningful stimulus. It is sensitive not only to semantic and linguistic processing^16–18^, but also to communicative gestures^19^, to the perception of images that are incongruent with context and expectations, or to violations of world-knowledge of the observer^20–24^.

In a recent study^25^, ERPs were recorded while participants viewed full-body pictures of actors displaying a particular emotional state, which could be congruent or incongruent with a preceding verbal description. The results showed that incongruent images elicited an anteriorly distributed N400, reflecting the recognition of incongruent affective body language. The EEG source analysis technique applied to Incongruent - Congruent condition difference wave in the N400 time window, showed, among others, the activation of the cingulate cortex, of the cortical areas underpinning face and body processing (STS, Fusiform Face Area-FFA and EBA) and of the PMc, which is involved in action comprehension and representation. Of note, these findings are in accord with the above mentioned three networks proposed for EBL processing^12^.

In the study by Proverbio *et al*.^25^, stimuli were full-body images (i.e., face was shown as a part of the whole body). Even though human bodies and faces are usually perceived as a whole and integrated in a unified percept, they should be considered as two different categories of stimuli, since their processing is underpinned by distinct (although strictly linked) neural networks^5^.

So far, electrophysiological investigation has been mainly focused on cerebral mechanisms for face and emotional facial expression comprehension^26–28^. On the contrary, few studies explored the time course and the neural correlates of EBL^25^, and even less investigated the integrative process between face and body emotion-related information^15, 29^.

In particular, using as stimuli face-body compound images, displaying identical or different emotional expressions, two recent ERP studies^15, 29^ have shown the influence of EBL on the perception of facial expressions, demonstrating an early integration of affective information between faces and bodies. Nonetheless, these studies focused just on the modulation of early ERP components, and did not explore the neural generators involved.

Given this background, the aim of the present study was to further investigate both the time course and the neural correlates underlying the integration of affective information conveyed by faces and bodies. To this purpose, high-density EEG was recorded during an expression matching task in a Congruence-Incongruence paradigm (N400 paradigm). Hence, differently from previously cited ERP studies aiming to investigate the influence of EBL on facial expressions perception^15, 29^, our stimuli were sequentially and not simultaneously presented, asking participants whether or not they displayed the same (congruent condition) or different (incongruent condition) emotion. We did not ask participants to explicitly judge the expression of any of our stimuli. Hence, we chose an expression matching task because, as stated by Bobes and colleagues (2000), it is capable to elicit “… *negative components that are enhanced for mismatches* … *and any type of material stored in long-term memory can elicit these negativities if the retrieved item is not integrated successfully with the context*”^30–35^. As far as we know from the literature, these paradigms have been mainly employed to investigate the time course of both the affective and semantic integration between faces^30–36^.

In our study, experimental stimuli were ecological pictures of facial and bodily expressions, both depicting two basic emotions: sadness and happiness. In two different sessions, participants judged the emotional congruence between sequentially presented pairs of stimuli (S1 and S2) belonging to the same category (face-face or body-body), and between stimuli belonging to different categories (face-body or body-face), respectively.

Through the analysis of the first session, we assessed the time course of the affective integration within a category and the neural networks involved. The analysis of the second session allowed us to better investigate the integrative mechanisms of face and body emotion-related information.

Focusing on N400 time window, we expected to find a significant modulation due to the emotional congruence of stimuli in all the conditions, irrespective of stimulus category. Indeed, in our hypothesis both emotional facial expressions and emotional body postures can create a powerful semantic and affective context, whereby participants could make judgment of congruence on a following emotional stimulus.

## Materials and Methods

### Participants

Twenty-four young adult volunteers took part in the study: ten male, 14 female; mean age 25.4 years (standard deviation, *SD* = 3.42); age range: 20–34 years. All participants had normal or corrected-to-normal visual acuity, no history of psychiatric or neurological impairments and were right-handed, as assessed by the Edinburgh Handedness Inventory^37^. Since data of four participants were discarded because of too many artefacts, data from twenty volunteers (nine male, 11 female; mean age 25.5 years (standard deviation, *SD* = 3.48); age range: 20–34 years) were included in the EEG analyses. All participants provided a written informed consent to participate in the study, which had been approved by the local ethical committee (Comitato Etico per Parma, Azienda Ospedaliero-Universitaria di Parma, Azienda Unità Sanitaria Locale di Parma, Università degli Studi di Parma) and has been conducted according to the principles expressed in the Declaration of Helsinki.

### Stimuli and Procedure

#### Stimuli

Stimuli were 32 previously validated pictures (16 bodies and 16 faces), displaying four actors (two males and two females) showing two basic emotions: sadness and happiness (for details regarding validation process, see Supplementary Materials). For each actor we selected two different postures for each emotional condition. Hence, stimulus material included eight happy (four male) and eight sad (four male) body postures, eight happy (four male) and eight sad (four male) facial expressions. By means of Adobe Photoshop CS6 software, pictures were placed at the centre of a homogenous grey background (R:128, G:128, B:128) reduced to a final dimension of 1280 × 1024 pixels. Stimuli were shown at the centre of a 19-inches computer screen positioned at a distance of 57 cm from participants. All faces and bodies had the same height of 4.70 cm, subtending 4.70° of visual angle, and different width to maintain proportions constant.

**Figure 1:**
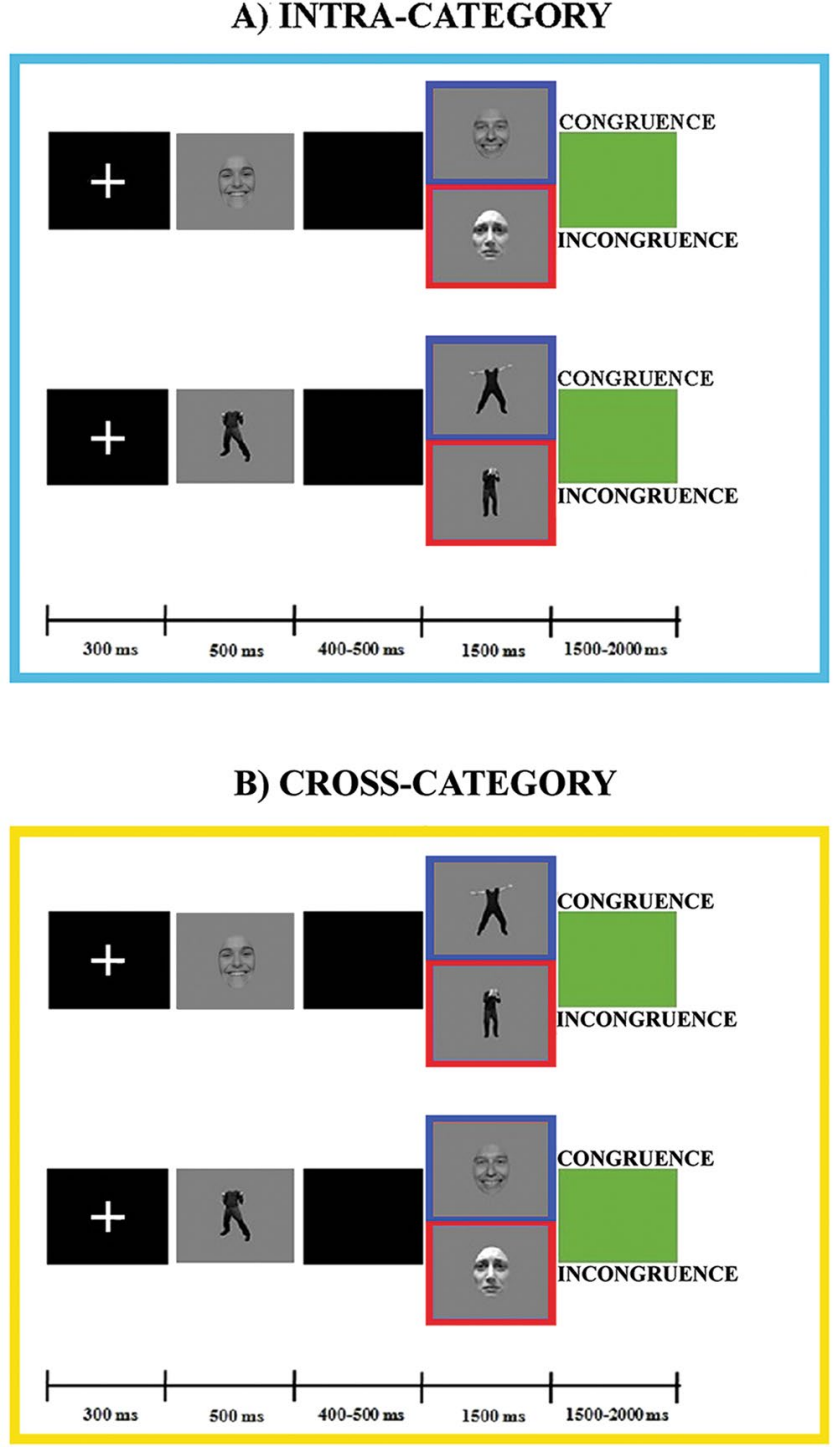
Experimental paradigm and stimuli.

The experimental paradigm included two different experimental sessions (“intra-category” and “cross-category”) and each session showed a two-picture sequence in two different experimental conditions (Congruent and Incongruent conditions). In the intra-category session, the first (S1) and the second (S2) stimulus pertained to the same category (i.e., face-face or body-body), while in the cross-category session they pertained to different categories (i.e., face-body or body-face). In both sessions, in the Congruent condition S1 and S2 displayed the same emotion (happiness-happiness or sadness-sadness), while in the Incongruent condition the emotion displayed was different (happiness-sadness or sadness-happiness) (Fig. 1). In sum, we had eight different conditions: face-face Congruent (FF-C), face-face Incongruent (FF-I), body-body Congruent (BB-C), body-body Incongruent (BB-I), face-body Congruent (FB-C), face-body Incongruent (FB-I), body-face Congruent (BF-C), body-face Incongruent (BF-I).

Each trial consisted of a white fixation cross (300 ms), followed by S1, which was presented for 500 ms. After an interstimulus interval of 400 or 500 ms, S2 was shown for 1500 ms, followed by a green screen used as intertrial interval with a duration of 1500 or 2000 ms (Fig. 1).

Each of the two sessions included 320 trials, divided in two sequences of 160 randomized trials.

Each picture was repeated 17–23 times per session, and repetition of the same stimulus within a trial was avoided. Hence, we had 80 trials per condition for a total of 640 trials. Each sequence lasted about 12 minutes, with a rest period of five minutes between each sequence. The order of presentation of the two sessions was balanced among participants.

#### Procedure

The task consisted of responding as accurately and quickly as possible by pressing a response key with the index finger (left or right hand) to S2 stimuli judged to be emotionally congruent to S1 and with the middle finger (left or right hand) to S2 stimuli judged to be incongruent. The hand used was alternated during the session (to avoid possible biases due to the prolonged activation of the contralateral hemisphere^25^) and hand order was counterbalanced across subjects. At the beginning of each sequence, the subjects were told which hand would be used to indicate their responses. To keep the attentional level of participants high, errors, omissions and delay (more than 1400 ms after stimulus onset) in responses were indicated through a beep sound lasting 200 ms (Fig. 1).

The experimental sessions were preceded by a training session including 16 randomized trials (eight intra-category and eight cross-category trials).

Stimuli delivery and response recording were controlled using E-prime 2.0 software.

### EEG Recording and Preprocessing

Continuous EEG was recorded using the 128-channels Geodesic EEG System (Electrical Geodesics Inc., Oregon) and the HydroCel Geodesic Sensor Net (GSN300), at a sampling rate of 500 Hz with the vertex as online reference; electrodes impedances were kept below 50 kΩ. Continuous EEG recordings were band-pass filtered (1–30 Hz) and segmented in epochs lasting 1100 ms (from 100 ms before to 1000 ms after S2 onset), by means of NetStation software (Electrical Geodesics, Inc., Eugene, OR, USA). Trials with omission and commission errors were excluded from further analysis. For artefact detection and removal, the epoch-file of each participant was imported in EEGLAB toolbox^38^.

**Figure 2:**
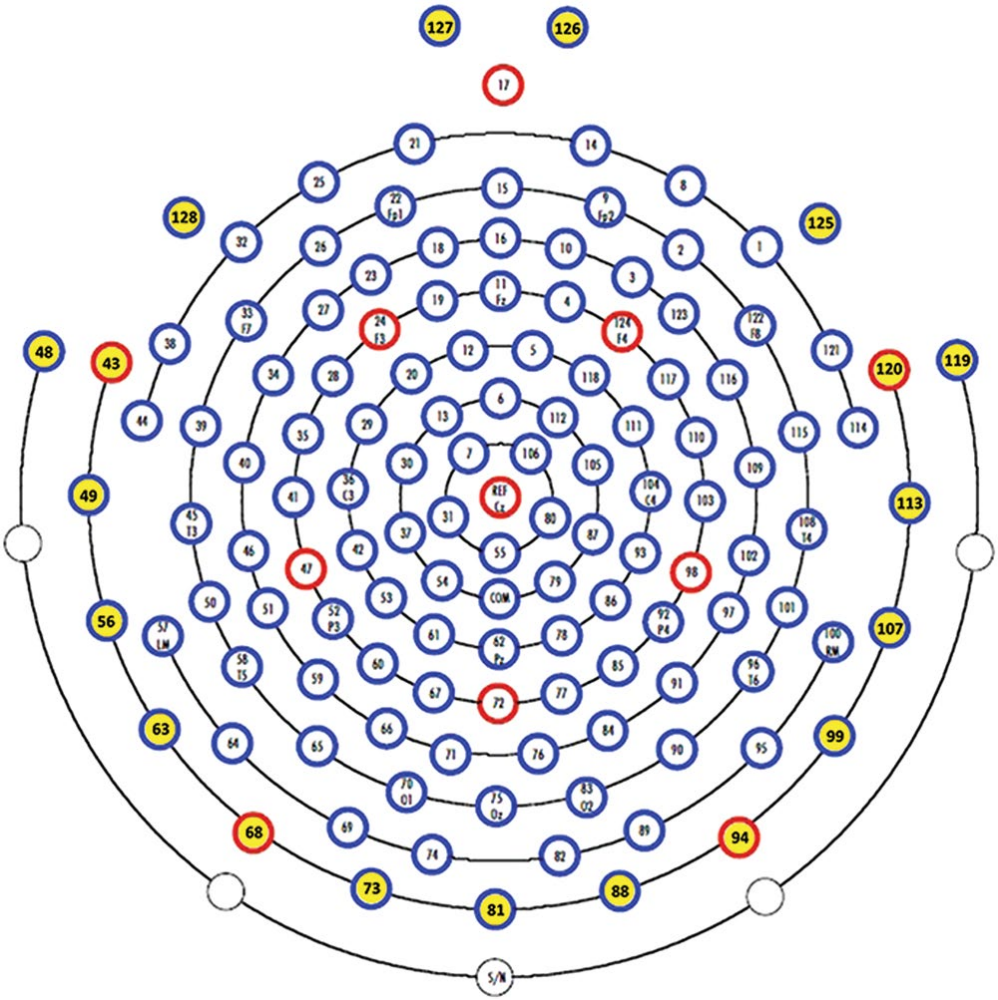
Hydrocel Geodesic Sensor Net – 128 channel map. In yellow the outermost belt of electrodes of the sensor net that was excluded from analyses.

The outermost belt of electrodes of the sensor net, more prone to show residual muscular artefacts, was excluded and the original template was reduced from 128 to 110 channels. Hence, we discarded 19 peripheral channels (E43, E48, E49, E56, E63, E68, E73, E81, E88, E94, E99, E107, E113, E119, E120, E125, E126, E127, E128) mainly located on the cheeks and on the nape and we then renamed the remaining 110 electrodes “e1–e110” (see Fig. 2). Epoch-files were then analysed by means of Independent Component Analysis (ICA)^38^, to exclude components bearing ocular and cardiac artefacts. A mean number of 8.1 (*SD* = 1.9) components were removed. The resulting epochs were further visually inspected to exclude residual artefacts. Bad channels were interpolated using a spherical interpolation method as implemented in EEGLAB, and recalculated against the average reference.

The mean of accepted trials for intra-category conditions was: 71.2 (*SD* = 3.4) for FF-C and 70.2 (*SD* = 5.4) for FF-I, 69.9 (*SD* = 5.4) for BB-C and 69.9 (*SD* = 3.7) for BB-I. The mean of accepted trials for cross-category conditions was: 70.9 (*SD* = 3.3) for FB-C and 70.8 (*SD* = 4) for FB-I, 70.4 (*SD* = 4.3) for BF-C and 69.2 (*SD* = 4.2) for BF-I.

An ANOVA was performed in order to exclude differences in the number of accepted trials among conditions, which did not result significant (F_(7, 133)_ = 1.203; *p* = 0.305).

Preprocessed data of each participant were subsequently imported and analysed in Cartool software (version 3.55; http://www.fbmlab.com/cartool-software/). To evaluate S2-elicited ERPs, epochs from 100 ms before to 1000 ms after S2 onset were averaged across trials, separately for each participant and condition; these single-participant averages were then used to compute eight group-averaged ERPs, one for each experimental condition.

#### EEG Analysis

S2-elicited EEG data were subjected to two analytic procedures, a global ERP waveform analysis and a global scalp electric field analysis. For the sake of completeness, the global ERP waveform analysis was performed as a preliminary step in determining the time course of ERP response modulations^39, 40^. It was conducted by means of point-wise paired *t* tests computed on amplitudes of the single-subject ERP averages of the two compared conditions (see below “Compared conditions” section), at each electrode and time point. The statistical significance level was set at *p* < 0.05 and a 10 contiguous data points temporal criterion (20 ms at our 500 Hz sampling rate) for the persistence of significant effects was applied^41^. Only differences over at least five contiguous electrodes within nine clusters (shown in the inset in Supplementary Figs S1 and S2) reaching the statistical significance level were retained.

The global scalp electric field analysis has two main advantages: 1) it allows the statistical assessment of the likely neurophysiological mechanisms (i.e., topographic and/or strength modulation) underlying the observed effect; 2) it allows one to define time windows for source analysis in a more objective way, relying on the statistical proof that when the electric fields are different they are underpinned by different neural generators^39^.

The global scalp electric field analysis approach is extensively described in previous papers^39, 42^ (see also Supplementary Materials). In brief, statistical analyses were conducted on two parameters: (1) the Global Field Power (GFP), measuring electric filed strength modulations; (2) the global spatial dissimilarity index (DISS), assessing modulations in electric field topography^42^. Modulations in GFP and DISS between experimental conditions were assessed by non-parametric statistical analyses based on point-wise randomization tests^43^. In the present study, the point-wise randomization tests ran 1000 permutations per data point and the significance level was set at *p* < 0.05, with an additional temporal stability acceptance criterion of 20 ms of consecutive significant difference^41^. Point-wise paired randomization performed on the DISS data is also known as “topographic analysis of variance” (TANOVA)^39^. The results of the TANOVA defined time periods during which intracranial sources were estimated, using a distributed linear inverse solution based on a Local Auto-Regressive Average (LAURA) regularization approach^44^. Intracranial source estimations for each participant and condition over time windows defined by the TANOVA-significant were statistically compared by means of a “voxel-wise parametric mapping analysis”^45^. LAURA source estimations for each solution point, normalized by root mean square (RMS values), were then contrasted by means of paired *t* tests. Solution points with *p* values < 0.05 (*t*_(19)_ > 2.09/ < −2.09) were considered significant; in addition, a cluster threshold of at least 10 contiguous activated solution points was applied. Source and statistical analyses were performed using Cartool software^40^.

Since the main purpose of the present study was to investigate the temporal dynamics of the incongruence effect (N400), we performed global scalp electric field analysis (GFP and DISS) from 100 to 1000 ms after the S2 onset.

#### Compared conditions

Considering that our main interest was in the incongruence effect (N400), we compared Congruent and Incongruent conditions of each session: 1) FF-I vs. FF-C; 2) BB-I vs. BB-C; 3) FB-I vs. FB-C; 4) BF-C vs. BF-I.

For details regarding behavioural analysis, please see Supplementary Materials.

### Data availability

The data that support the findings of this study are available from the corresponding author upon reasonable request.

## Results

**Figure 3:**
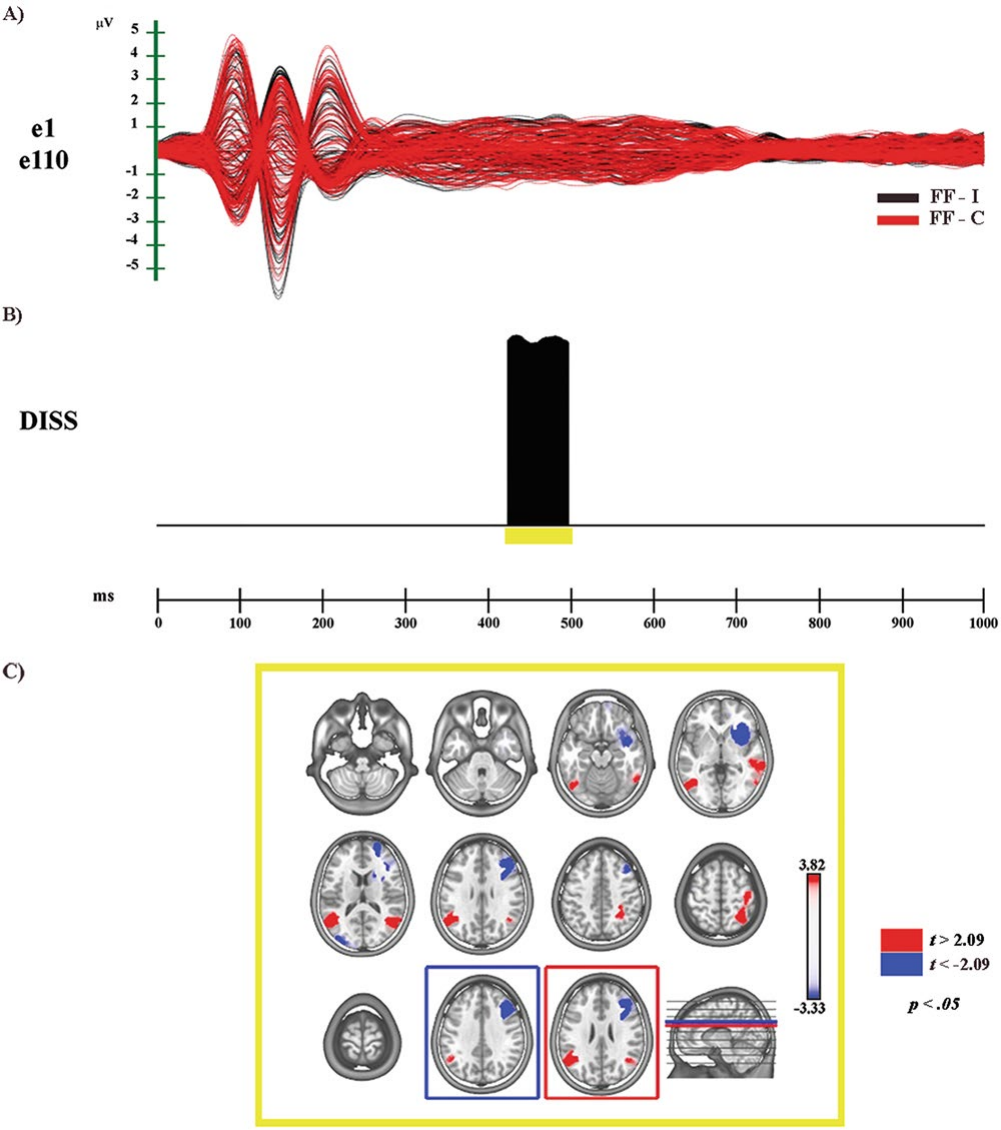
Electrophysiological results and statistical comparison of LAURA source estimations between FF-I and FF-C over significant TANOVA time interval (N400). (**A**) Group-averaged (n = 20) event related potential (ERP) waveforms of the two experimental conditions (FF-C and FF-I), superimposed across the 110 recording channels (e1–e110). Black: FF-I; red: FF-C. (**B**) Global scalp electric field analyses: statistical analysis of global electric field topography (topographic analysis of variance, TANOVA). Black area indicates time interval of significant differences (*p* < 0.05; duration ≥20 ms) of global spatial dissimilarity index (DISS). (**C**) Significant TANOVA time interval (424–496 ms after S2 onset). All significant voxels are colored (*t*_(19)_ > 2.09/< −2.09, p < 0.05): positive *t* values (red color) indicate higher current source densities in FF-I than in FF-C; negative *t* values (blue color) indicate higher current source densities in FF-C than in FF-I. LAURA solutions are rendered on MNI152 template brain (left hemisphere on the left side).

**Figure 4:**
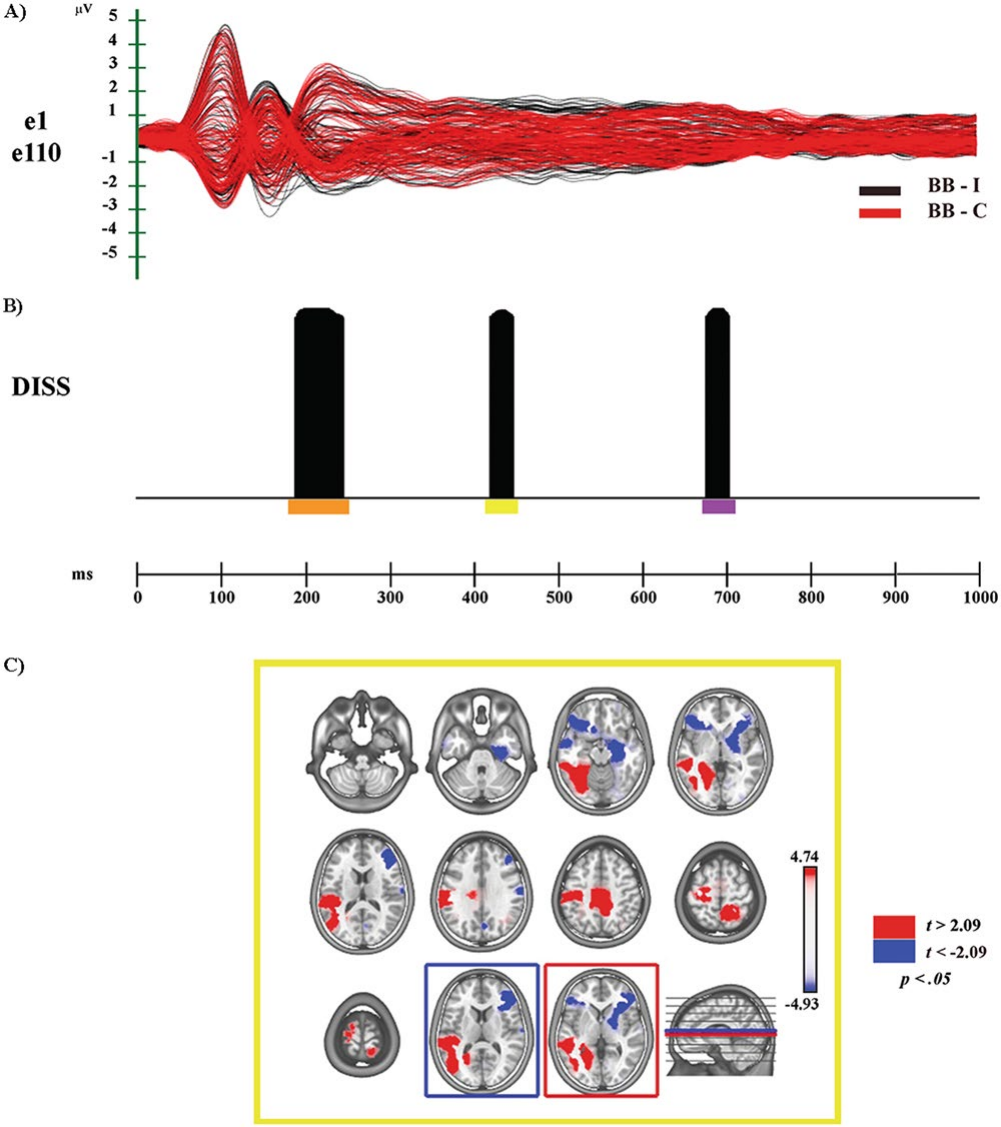
Electrophysiological results and statistical comparison of LAURA source estimations between BB-I and BB-C over significant TANOVA time interval (N400). (**A**) Group-averaged (n = 20) event related potential (ERP) waveforms of the two experimental conditions (BB-C and BB-I), superimposed across the 110 recording channels (e1–e110). Black: BB-I; red: BB-C (**B**) Global scalp electric field analyses: statistical analysis of global electric field topography (topographic analysis of variance, TANOVA). Black areas indicate time intervals of significant differences (*p* < 0.05; duration ≥20 ms) of global spatial dissimilarity index (DISS). (**C**) Significant TANOVA time interval (418–446 ms after S2 onset). All significant voxels are colored (*t*_(19)_ > 2.09/< −2.09, p < 0.05): positive *t* values (red color) indicate higher current source densities in BB-I than in BB-C; negative *t* values (blue color) indicate higher current source densities in BB-C than in BB-I. LAURA solutions are rendered on MNI152 template brain (left hemisphere on the left side).

**Figure 5:**
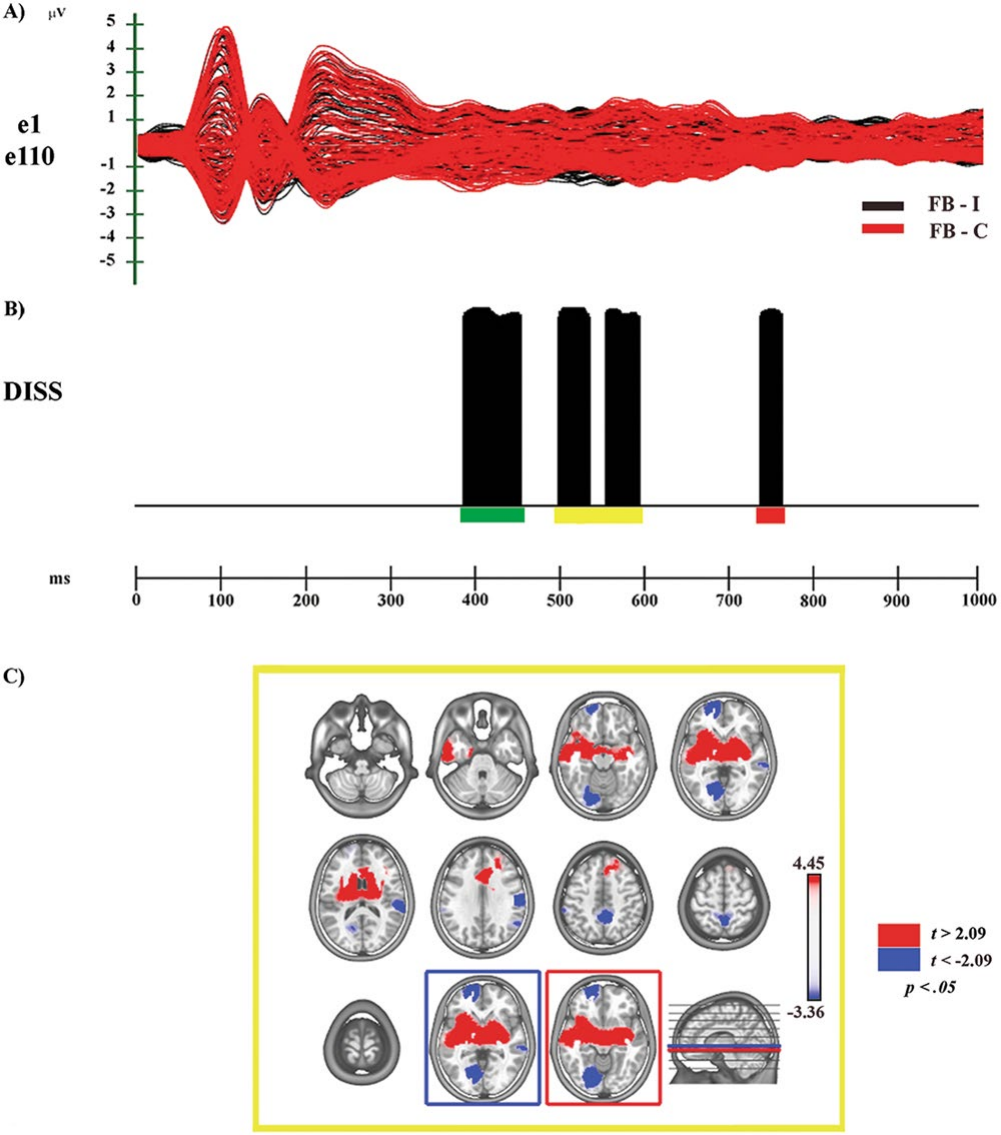
Electrophysiological results and statistical comparison of LAURA source estimations between FB-I and FB-C over significant TANOVA time interval (N400). (**A**) Group-averaged (n = 20) event related potential (ERP) waveforms of the two experimental conditions (FB-C and FB-I), superimposed across the 110 recording channels (e1–e110). Black: FB-I; red: FB-C (**B**) Global scalp electric field analyses: statistical analysis of global electric field topography (topographic analysis of variance, TANOVA). Black areas indicate time intervals of significant differences (*p* < 0.05; duration ≥20 ms) of global spatial dissimilarity index (DISS). (**C**) Significant TANOVA time interval (498–594 ms after S2 onset). All significant voxels are colored (*t*_(19)_ > 2.09/< −2.09, p < 0.05): positive *t* values (red color) indicate higher current source densities in FB-I than in FB-C; negative *t* values (blue color) indicate higher current source densities in FB-C than in FB-I. LAURA solutions are rendered on MNI152 template brain (left hemisphere on the left side).

The electrophysiological results of global ERP waveform analyses and scalp electric field analyses, and source estimations in the N400 time period, are reported separately for each comparison (see Figs 3–5 and Supplementary Figs S1 and S5).

For more details about global amplitude analysis, source estimations in other ERP significant time periods, and for behavioural results, see Supplementary Results and Supplementary Figures S1–S7.

### FF-I vs. FF-C

#### Electrophysiological Results

The global amplitude analysis (see Supplementary Fig. S1A) revealed three periods of significant ERP modulation: (1) from 132 to 196 ms, compatible with a N170 modulation of higher amplitude to Incongruent than to Congruent condition; (2) from 200 to 250 ms, similar to a fronto-central N200 of higher amplitude in response to Congruent than to Incongruent condition; (3) from 418 to 464 ms after S2 onset, identified as N400 modulation of higher amplitude to Incongruent than to Congruent condition (see Supplementary Fig. S2). The GFP analysis (see Supplementary Fig. S1B) showed one period of sustained difference between conditions, from 136 to 160 ms after S2 onset. The TANOVA (see Fig. 3B) revealed one phase of significant topographic difference between conditions, from 424 to 496 ms after S2 onset.

In summary, GFP and DISS analyses revealed that, while the first period of amplitude modulation (132–196 ms after S2 onset) was characterized by strength difference between conditions, different cerebral generators underpinned only the third period (418–464 ms after S2 onset).

#### Source estimations

For the time period of different topography (424–496 ms after S2 onset) significant higher activity in FF-I as compared with FF-C (see Fig. 3C, in red; Table 1) was found in different cerebral regions, including: the left middle temporal gyrus (MTG) extending toward the supramarginal gyrus (BAs 19, 37, 39, 40) and the right temporal (BA 39) and parietal cortices with an involvement of associative somatosensory regions (BAs 5, 7, 40). Higher activity in FF-C (see Fig. 3C, in blue; Table 1) was found in right middle and superior frontal gyri (BAs 8, 9, 46) and in right IFG and adjacent anterior insula (BAs 13, 46, 47).

**Table 1:** Source localization of topographic maps.

Condition	TANOVA N400 time period	*t* value	*p* value	Talairach coordinates (x, y, z) mm	Brain region label
FF-I > FF-C	424–496 ms	3.82	0.001	−48, −53, 27	Left superior temporal gyrus, BA^1^ 39
		2.64	0.01	48, −54, 20	Right superior temporal gyrus, BA 39
		2.33	0.03	33, −45, 47	Right precuneus, BA 7
FF-C > FF-I		−3.33	0.003	33, −19, 30	Right middle frontal gyrus, BA 9
		−3.30	0.003	33, 18, 2	Right insula
BB-I > BB-C	418–446 ms	4.74	0.0001	−41, −32, 5	Left superior temporal gyrus, BA 41
		3.84	0.001	−56, −16, 32	Left postcentral gyrus, BA 3
		3.25	0.004	11, −45, 54	Right precuneus, BA 7
		2.75	0.01	−18, −31, 39	Left posterior cingulate gyrus, BA31
BB-C > BB-I		−4.93	0.00009	33, 33, 8	Right inferior frontal gyrus, BA 46
		−3.45	0.002	33, −11, −15	Right parahippocampal gyrus
		−3.04	0.002	−33, 32, −4	Left inferior frontal gyrus, BA 47
FB-I > FB-C	498–594 ms	4.45	0.0003	41, −11, −8	Right temporal sub-gyral, BA 21
		4.17	0.005	−41, −11, −2	Left insula, BA 13
FB-C > FB-I		−3.36	0.009	−11, −69, 0	Left lingual gyrus, BA 18
		−3.26	0.004	63, −25, 4	Right superior temporal gyrus BA 22
		−3.20	0.005	−11, 62, −6	Left medial frontal gyrus, BA 10
		−3.15	0.005	3, −45, 54	Right precuneus, BA 7

Significant results of the statistical comparisons of LAURA source estimations in significant TANOVA N400 time periods are reported, with *t* and *p* values, Talairach and Tournoux coordinates (x, y, z) and anatomical labels of solution points with the local maximum different activities. BA = Brodmann Area.

### BB-I vs. BB-C

#### Electrophysiological Results

The global amplitude analysis (see Supplementary Fig. S1C) revealed three periods of significant ERP modulation: (1) from 138 to 274 ms, compatible with a fronto-central N200 modulation, of higher amplitude in response to Congruent than to Incongruent condition; (2) from 370 to 554 ms, identified as N400 component, of higher amplitude for Incongruent than for Congruent condition; (3) from 682 to 706 ms after S2 onset, compatible with a posterior-central Late Positivity (LP) of higher amplitude in response to Congruent than to Incongruent condition (see Supplementary Fig. S3). The GFP analysis (see Supplementary Fig. S1D) showed five periods of sustained difference between conditions: (1) from 128 to 216 ms; (2) from 252 to 326 ms; (3) from 406 to 556 ms; (4) from 898 to 942 ms; (5) from 970 to 1000 ms after S2 onset. The TANOVA (see Fig. 4B) revealed three periods of significant topographic difference between conditions: (1) from 188 to 244 ms, yellow bar; (2) from 418 to 446 ms, orange bar; (3) from 676 to 702 ms after S2 onset, purple bar.

In sum, GFP and DISS analyses revealed that the first (138–274 ms) and the second (about 370–554 ms) periods of amplitude modulation were characterized by both strength and topographic differences between conditions. The third period (682 to 706 ms) was underpinned by different cerebral generators.

#### Source estimations

In the second significant TANOVA period (418–446 ms after S2 onset) higher activity in BB-I (see Fig. 4B - yellow bar; see Fig. 4C, in red) was found in different cerebral regions (Table 1), including left occipitotemporal areas for visual body processing, encompassing the MTG (BAs 18–22, 36, 37, 39, 42) and left premotor and motor cortices with the involvement of the IPL (BAs 4, 6, 40). Moreover, on the right hemisphere it is noteworthy the significant activation of somatosensory cortices (BAs 3–5, 7). In the same time period, higher activity in BB-C condition (see Fig. 4C, in blue; Table 1) was found in bilateral IFG (BAs 46, 47).

### FB-I vs. FB-C

#### Electrophysiological Results

The global amplitude analysis (see Supplementary Fig. S5A) revealed six periods of significant ERP modulation: (1) from 194 to 250 ms, compatible with a central N200 modulation, with higher amplitude to Congruent than to Incongruent condition; (2) from 372 to 456 ms, compatible with a central P300 modulation of higher amplitude to Congruent than to Incongruent condition; (3) from 496 to 568 ms; (4) from 580 to 608 ms; these two windows are compatible with an extended N400 modulation, of higher amplitude to Incongruent than to Congruent condition (5) from 626 to 716 ms (6) from 726 to 786 ms after S2 onset, considered as a LP modulation with higher amplitude to Congruent than to Incongruent condition (see Supplementary Fig. S6). The GFP analysis (Supplementary Fig. S5B) showed two periods of sustained difference between conditions: (1) from 514 to 540 ms; (2) from 652 to 688 ms after S2 onset.

The TANOVA (see Fig. 5B) revealed four phases of significant topographic difference between conditions: (1) from 386 to 454 ms, green bar; (2) from 498 to 534 ms, yellow bar; (3) from 552 to 594 ms, yellow bar; (4) from 736 to 762 ms after S2 onset, red bar.

In summary, GFP and DISS analyses revealed that the second (370–456 ms), the fourth (between 580–608 ms) and the sixth (726–786 ms) periods of amplitude modulation were characterized by topographic differences between conditions. The fifth period (630–716 ms) of amplitude modulation was characterized only by strength modulation, while the third (496–568 ms) period of amplitude modulation was characterized by both strength and topographic differences between conditions.

Since the interval between the second and the third phases was shorter than the temporal acceptance criterion of consecutive 20 ms of significant difference, we estimated the intracranial sources of second and third phases together (from 498 to 594 ms).

#### Source estimations

In the second significant TANOVA period (498–594 ms after S2 onset) higher activity in FB-I (see Fig. 5B – yellow bar; Fig. 5C, in red; Table 1) was found in different areas including: bilateral IFG (BAs 46, 47) and ACC (BAs 24, 32, 33), left PMc (BA 6) and right MTG and STS (BAs 21, 22). Higher activity in FB-C (see Fig. 5C, in blue; Table 1) was found, among others, in right somatosensory-related cortices and IPL (BAs 1, 3, 40) extending towards MTG and STS (BAs 22, 41, 42).

### BF-I vs. BF-C

#### Electrophysiological Results

The global amplitude analysis (see Supplementary Fig. S5C) revealed three periods of significant ERP modulation: (1) from 54 to 102 ms, compatible with an anterior N100 of higher amplitude to Incongruent than to Congruent condition; (2) from 332 to 374 ms, identified as a P300 modulation of higher amplitude to Congruent than to Incongruent condition; (3) from 656 to 692 ms after S2 onset, compatible with a central-posterior LP modulation of higher amplitude to Congruent than to Incongruent condition (see Supplementary Fig. S7). The GFP analysis and the TANOVA did not show periods of sustained difference between conditions.

## Discussion

The aim of the present study was to investigate the time course and neural correlates of the integration of body and face emotion-related information. Specifically, we aimed at clarifying similarities and differences between face and body emotional processing and to assess how the affective integrative process of emotional faces and bodies is built up.

To this purpose we used as stimuli ecological pictures of facial and bodily expressions depicting sadness and happiness. We recorded electrophysiological indexes while participants judged the emotional congruence between pairs of sequentially presented stimuli belonging to the same category (face-face, body-body), or to different categories (face-body, body-face).

Our results confirmed the presence of a modulation of the N400, with higher amplitude in response to incongruent than to congruent stimuli, in all comparisons except for body-face condition, where we did not find significant difference between Congruence (BF-C) and Incongruence (BF-I).

For reasons of clarity, here we focus on the N400 results: for an extensive discussion regarding ERP modulations in other time periods, see Supplementary Materials. First we discuss the results of each comparison, and then we conclude with a general discussion.

### FF-I vs FF-C

In accord with previous literature, our results showed higher N400 amplitude in response to the FF-I than to the FF-C condition, as indexed by a significant period of amplitude modulation between 420 and 460 ms after S2 onset. The global scalp electric field analysis revealed that this modulation was characterized by topographic differences between the two conditions. Significant higher activity in FF-I was found in bilateral MTG and STS extending toward IPL in the left hemisphere, with an involvement of somatosensory association regions in the right one (Fig. 3C). Regarding MTG, previous studies showed its involvement in both emotional face perception and discrimination of expressive faces^27, 46^. In our results the bilateral MTG was highly engaged in the Incongruent condition: this data suggest a further role of the MTG in the storage and interpretation of stimulus meaning, in line with the integrative hypothesis for the N400 effect^17^.

The activation of the STS together with bilateral parietal regions could index the involvement of the MM for emotion recognition during the processing of FF-I condition^47^. In this regard, it has been hypothesized that the right somatosensory cortex could contribute to the visual recognition of emotional facial expression. By means of an embodied simulation mechanism of “… *how one would feel if making the facial expression shown in the stimulus*”^48^ this cerebral region would provide the information needed to understand the meaning of the observed expression^49–51^.

In contrast, during the FF-C condition processing our data revealed activation of areas (such as the inferior frontal cortex and the adjacent insula, Fig. 3C) pertaining to the cerebral “extended system” for face perception, devoted to processing of changeable facial aspects^52–55^.

Taken together, these results suggest that on the one hand, the “extended system” for face perception would be sufficient for the processing of emotion when S1 and S2 conveyed congruent information. On the other hand, the access to the meaning storage system and the activation of sensorimotor representations are needed during the processing of the Incongruent condition, in order to solve the conflict created by the incongruence between S1- and S2-related affective information.

### BB-I vs BB-C

In the second TANOVA significant time period (418–446 ms), corresponding to the N400 modulation, higher activity in BB-I emerged in left occipitotemporal regions of the ventral stream for body processing. As for incongruent faces processing, higher activity was also found in left MTG and STS (Fig. 4C), regions that have been related to storage and interpretation of stimulus meaning^17^, and to the analysis of movement and biological actions^24, 56^, respectively. Furthermore, higher activation of left motor (M1) and premotor cortices together with the IPL, and of left primary (SI) and bilateral associative somatosensory cortices emerged during BB-I condition (Fig. 4C).

In this regard, M1 and PMc are tipically considered part of the MM for action, while somatosensory-related cortices were shown to contribute to the perception of others and of their actions^48, 57^.

In BB-C condition we found higher activity in bilateral IFG and right limbic regions (Fig. 4C). These activations could be related to the processing of semantic/affective aspects of bodies as in the “extended system” for faces^52–55^.

In sum, our findings are in accord with the model of emotion-behaviour connectivity for EBL comprehension proposed by de Gelder^12^, showing the activation of regions for the visuomotor perception of EBL. Indeed, the major finding of our study is the involvement of motor, premotor and somatosensory regions during the processing of the Incongruent condition, especially in the N400 time window. Hence, the resolution of the conflict between information conveyed by the S1 and S2 requires the retrieving of sensorimotor representations during the processing of emotional body postures.

### FB-I vs FB-C

In the second significant TANOVA time period (498–594 ms), corresponding to the N400 modulation, during FB-I condition higher activity emerged in right MTG, STS and in bilateral ACC (Fig. 5C). Of note, the activation of MTG and STS emerged also in the N400 time window of the other Incongruent conditions (FF-I, BB-I), suggesting their role in emotion-related processing for both faces and bodies. Furthermore, the activation of bilateral IFG and PMc (Fig. 5C) could represent the involvement of the MM for action, underpinning the processing of motor information conveyed by images of body postures expressing emotions.

Of note, in this experimental condition (and in BF-I vs. BF-C too), FB-I contained a two-level conflict: a first level due to a perceptual incongruence (S1 and S2 pertained to different stimulus categories) and a second level due to the emotional incongruence. This peculiarity could explain the higher activation of somatosensory-related regions in FB-C: despite the absence of an emotional incongruence, this condition is still characterized by the perceptual incongruence between S1 and S2 (face vs. body). Possibly, by means of an embodied simulation mechanism these regions would have provided the information needed to comprehend the meaning of S1 (facial expression)^48, 50, 51^ (see also FF-I vs. FF-C section). As a consequence, the involvement of the same regions during S2 perception (bodily expression) was needed to link the two emotional information and to solve the task.

In sum, our findings suggest that the affective integration of emotional faces and bodies is build up through the activation of a MM for action and emotion comprehension, but with the involvement of different regions according to the emotional Congruence/Incongruence condition. In the presence of both perceptual and emotional conflict (FB-I), it involves a more action-related simulation mechanism, requiring also the access to the meaning storage system. Conversely, when the conflict is only at a perceptual level (same emotion condition, FB-C) the simulation mechanism seems to be more somatosensory related.

### BF-I vs BF-C

Although the global amplitude analysis revealed three periods of amplitude modulation, the global scalp electric field analyses revealed that this modulation was characterized neither by strength nor by topographic differences between conditions. This could suggest that emotional body postures were not effective as contextual stimuli in creating a clear emotional context whereby participants could make judgment of congruence on a following emotional facial expression. Future studies with large experimental samples will be needed to further assess this condition.

### General discussion and conclusions

The current results confirm previous findings regarding emotional facial expression and EBL comprehension mechanisms^12, 25, 47, 48^. Furthermore, they provide new evidence for the presence of both similarities and differences between face and body emotional processing.

During the N400 time window, “intra-category” session results showed that in the presence of emotional incongruence between S1 and S2, both access to the meaning storage system (likely represented by the MTG) and activation of an embodied simulation mechanism by means of the MM, were necessary to solve the conflict between S1- and S2-related affective information. Specifically, while the perception of incongruent facial expressions activates somatosensory-related representations, incongruent emotional body postures would also require the retrieval of motor and premotor representations, in the perspective of a more strictly link between emotion and action during the processing of incongruent emotion conveyed by bodies with respect to faces^58^.

Conversely, when S1- and S2-related emotional information were congruent, an involvement of inferior frontal and limbic regions emerged during the processing of both facial expressions and emotional body postures. These findings point to a pivotal role of the “extended system” for the elaboration of semantic/affective aspects not only for faces, as previously suggested^52–55^ but also for body postures and EBL.

The analyses of “cross-category” session allow us to assess how the integrative process of emotional faces and bodies is built up. Our findings during the N400 time window of FB-I vs. FB-C comparison revealed that faces were able to build up a clear affective context as S1 stimuli, so that the affective integration process could ensue, allowing a judgement of congruence, by accessing the meaning storage system and activating the MM for action.

In this regard, the MM had a role during both Congruent and Incongruent conditions, but with different patterns of activity. Specifically, the MM activated brain regions mainly action-related during FB-I, and more somatosensory-related during FB-C condition. Of note, contrary to what happens when faces were used as S1, emotional body posture were unable to trigger a semantic/affective integration mechanism. Indeed, BF-I vs. BF-C comparison did not show significant modulation of the N400. Possibly, these negative findings are due to the specific emotional body postures that we used in the present study. Whether other emotions (e.g. fear and anger) could be more powerful in creating a clear emotional context remains an open issue for future research.

Moreover, we did not investigate possible modulatory effects of the chosen emotions in the intra-category conditions, or the influence of the order of emotions in the cross-category conditions. Taking into account the related recent evidence from de Borst and de Gelder (2016)^59^, we think this could be considered as a limitation of our study and should be addressed with a future study, capable to make the puzzle clearer.

Additionally, bearing in mind that the high-density EEG has an optimal temporal resolution but a spatial resolution not comparable to other neuroimaging techniques (e.g., fMRI), future studies are needed to assess the reliability of our source level findings. More specifically, several methodological limitations should be taken into account while interpreting the source level results. They are mainly related to both the forward modelling and the inverse solution algorithms employed, as well as to the choice to use an MNI average template and not single-subject MRI scansions which could have improved the accuracy of source projection in the volumetric space.

In conclusion, the present results shed new light on EBL comprehension mechanisms, clarifying commonalities and differences with well-known facial expressions processing. Future studies should further assess the relevance of emotional body postures to create effective semantic and emotional contexts with sufficient adaptive information, in absence of other emotional cues (e.g. facial expressions).

## Electronic supplementary material

Supplementary Materials
