# Supplementary material for: “Embodied Body Language”: an electrical neuroimaging study with emotional faces and bodies
Source: Sci Rep. 2017 Jul 31;7:6875. doi: 10.1038/s41598-017-07262-0 (PMC5537350; doi:10.1038/s41598-017-07262-0)
Supplement: Supplementary file 1 — Supplementary Materials [file 41598_2017_7262_MOESM1_ESM.pdf]

"Embodied Body Language": an electrical neuroimaging study with emotional faces and bodies.

Marta Calbi[1*], Monica Angelini[2], Vittorio Gallese[1,3], Maria Alessandra Umiltà[4]

[1] Department of Medicine and Surgery, Unit of Neuroscience, University of Parma, Parma, Italy

[2] Department of Clinical and Experimental Sciences, University of Brescia, Brescia, Italy

[3] Institute of Philosophy, School of Advanced Study, University of London, UK

[4] Department of Food and Drug Sciences, University of Parma, Parma, Italy

* Corresponding author

E-mail: calbimarta@gmail.com

Stimuli validation

Stimuli were generated by taking ecological pictures of emotional body postures and facial expressions. Eight professional actors (four males; age range: 23-34 years) were asked to display two different emotional states (happiness and sadness) and a neutral condition using their entire body and their face.

Photographs were taken in a classroom while the actors stood in front of a digital camera mounted on a static tripod in a black hall in light-controlled conditions. A set of standardized instructions was given to each actor indicating which was the emotion to display.

For each emotional category, we took pictures of different postures resulting in a total of 338 pictures (172 pictures of faces and 166 pictures of entire bodies). By means of Adobe Photoshop CS6 software, faces were cropped to remove external facial features (e.g. hair) and bodies were

processed to remove the head. Each picture was then converted in grey-scale and presented on homogenous grey background (R:128, G:128, B:128).

To test the validity of the pictures (i.e., to ensure that they were easily comprehensible in terms of their intended emotions), they were presented to a group of 15 judges (seven men) with a mean age of 25.5 years (age range: 22-35 years).

Each picture was randomly presented for three seconds and participants were asked to rapidly categorize the emotion displayed by each image as "happiness", "sadness", "neutral" or "none of these". The option "none of these" has been given *to reduce the likelihood that agreement on a particular option was an artefact of the response format*[60,61]. Only pictures that were evaluated consistently by at least 80% of the judges were included in the experimental set. On the basis of the results obtained by the validation, we selected 32 pictures (16 bodies and 16 faces), displaying four actors (two male). The average percentage of recognition was 98% for emotional facial expressions and 95% for emotional body postures.

EEG Analysis

Global electric field analyses

Two statistical analyses were conducted on the global electric field: a) assessment of modulations in electric field strength, as measured by the instantaneous Global Field Power (GFP); b) assessment of modulations in electric field topography, measuring the global spatial dissimilarity index (DISS)[42].

Significant modulations in GFP and DISS between the compared experimental conditions were assessed by non-parametric statistical analyses based on point-wise randomization tests[43]. Randomization provides a robust non-parametric method to test for differences in any variable without any assumption regarding data distribution, by comparing the observed data set with

random shuffling of the same values over sufficiently large number of iterations (i.e., permutations); this method allows one to determine the probability that the data might be observed by chance. In the present study, the point-wise randomization tests ran 1000 permutations per data point and the significance level was set at $p < .05$, with an additional temporal stability acceptance criterion of 20 ms of consecutive significant difference[41].

These two analyses allowed a neurophysiological interpretation of the ERP modulations: indeed, differences in GFP without simultaneous topographic changes are indicative of amplitude modulation of statistically indistinguishable generators between experimental conditions. Conversely, topographic differences between conditions, with or without concomitant GFP modulations, necessarily derive from changes in the configuration of the underlying active brain sources[39].

Changes in electric field strength were assessed by means of the statistical comparison of the GFP between compared conditions for each participant[39,42]. GFP is the spatial standard deviation of the potentials at all electrodes at a given time point: it is calculated as the square root of the mean of the squared value recorded at each electrode (measured versus the average reference) and has higher values for stronger electric fields[39,42]. Point-wise paired randomizations were conducted on the GFP of single-subjects ERP averages between conditions at each time frame, with a significance level set at $p < .05$ and a temporal acceptance criterion of 20 ms of consecutive significant difference.

Significant periods of topographic modulation were identified using randomization statistics applied to DISS[39,42] between conditions, calculated for each time point and each participant data. DISS is a strength-independent index of configuration differences between two electric fields and it is calculated as the square root of the mean of the squared differences between the instantaneous voltage potentials (measured versus the average reference) across the electrodes montage, each of which is first scaled to unitary strength by dividing it by the instantaneous GFP. Point-wise paired randomizations were performed on the DISS data: this analysis is also known as "topographic

analysis of variance" (TANOVA) (Murray et al., 2008). As above, 1000 permutations for each time point were performed and only effects with $p < .05$ and lasting for 20 ms or longer[41] were considered significant.

While GFP modulations indicate quantitative changes, DISS modulations between sessions reflect qualitative changes in the underlying generators configuration[39].

The results of the above topographic global scalp electric field analysis (TANOVA) defined time periods during which intracranial sources were estimated, using a distributed linear inverse solution based on a Local Auto-Regressive Average (LAURA) regularization approach[44]. LAURA model reconstructs the brain electric activity in each point of a 3D grid of solution points, selecting the source configuration that better mimics the biophysical behavior of electric fields without *a priori* assumption on the number of dipoles in the brain. The solution space was calculated on a locally spherical head model with anatomical constraints (L-SMAC)[62] and comprised 3001 solution points (voxels) homogeneously distributed within the brain structures of the Montreal Neurological Institute (MNI152) average brain. All solution points were labeled with their Talairach and Tournoux coordinates[63] as well as their anatomical labels.

Intracranial source estimations for each participant and condition over time windows defined by the TANOVA were then statistically compared by means of a "voxel-wise parametric mapping analysis"[45]. To do that, individual ERP data were averaged over time periods of significant topographic modulation, in order to generate a single data point per period for each participant and condition.

LAURA source estimations for each solution point, normalized by their RMS values (root mean square), were then contrasted by means of paired *t* tests. Solution points with *p* values < .05 ($t_{(19)} > 2.09/ < -2.09$) were considered significant; in addition, a cluster threshold of at least 10 contiguous activated solution points was applied. Source analyses were performed using Cartool software[40].

Behavioural Analysis

Reaction times (RTs) that exceeded the mean value ±2 *SD* were discarded. Accuracy data were converted to arcsin values. Both RTs and accuracy data were subjected to separate multifactorial repeated-measures ANOVAs with eight within-subject factors (S1: face or body; S2: face or body; Condition: Congruence or Incongruence; Response hand: left or right). Tukey post hoc tests were used to further explore significant interactions.

Results

Behavioural Results

Analysis of accuracy data revealed a significant main effect of S2 ($F_{(1,23)}$ = 12.066, p = .002) indicating that participants were more accurate when S2 was a face (80.9, *SE* = 1.28) than when it was a body (78.8, *SE* = 1.13). Participants were more accurate in response to Congruent (80.5, *SE* = 1.1) than to Incongruent pictures (79.2, *SE* = 1.29) as indicated by a significant main effect of Condition ($F_{(1,23)}$ = 6.7091, p = .016). The ANOVA also revealed a significant S2 x Hand interaction ($F_{(1,23)}$ = 5.3396, p = .03) that was driven by more accuracy in responding to body-S2 with the right hand (79.8, *SE* = 1.03) than when participants responded with the left hand (77.8, *SE* = 1.32) (post hoc tests: p = .03). When participants responded to face-S2 there was no difference responding with right or left hand (post hoc tests: p = .98). The significant Condition x Hand interaction ($F_{(1,23)}$ = 7.5332, p = .01) revealed that in the Congruent condition participants made less error when they responded with the right hand (81.8, *SE* = 1.04) than when with the left hand (79.3, *SE* = 1.29) (post hoc tests: p = .0006). In the Incongruent condition, instead, there was no difference due to response hand (post hoc tests: p = .98). In sum, when participants responded with the right hand there was a significant difference in accuracy data between Congruence and Incongruence (Congruence: 81.8, *SE* = 1.04, Incongruence: 78.9, *SE* = 1.25) (post hoc tests: p = .008), but not

when they responded with the left hand (Congruence: 79.3, *SE* = 1.29; Incongruence: 79.6, *SE* = 1.50).

Analysis of the reaction times (RTs) revealed a main effect of S2 ($F_{(1,23)}$ = 25.841, p = .00004) that was due to the responses to face-S2 (702 ms, *SE* = 17.8) being faster than those to body-S2 (731 ms, *SE* = 17.6). The significant main effect of S1 ($F_{(1,23)}$ = 11.591, p = .002) was driven by faster responses when S1 was a face (711 ms, *SE* = 17.6) than when it was a body (723 ms, *SE* = 17.5). Participants were faster responding to Congruent (687 ms, *SE* = 17) than to Incongruent condition (746 ms, *SE* = 18.9) as revealed by a significant main effect of Condition ($F_{(1,23)}$ = 53.098, p = .000). The significant S2 x Condition interaction ($F_{(1,23)}$ = 18.406, p = .0002) revealed faster responses S2 in the Congruent condition (Face: 666 ms, *SE* = 17.7; Body: 708 ms, *SE* = 16.8) than responses to S2 in the Incongruent condition (Face: 739 ms, *SE* = 19; Body: 754 ms, *SE* = 19.2), with a significant difference among all conditions (post hoc tests: p < .01). The significant S1 x Condition interaction ($F_{(1,23)}$ = 12.457, p = .002) indicated absence of significant differences in RTs due to S1 (face or body) in the Incongruent condition (Face: 747 ms, *SE* = 19.3; Body: 745, *SE* = 18.8), while in the Congruent condition RTs were faster when S1 was a face (675 ms, *SE* = 16.7) than when it was a body (700 ms , *SE* = 17.6) (post hoc tests: p < .0007). The significant S1 x Hand interaction ($F_{(1,23)}$ = 5.4463, p = .03) revealed faster responses to S1 with the left hand (Face: 716.3 ms, *SE* = 20; Body: 736 ms, *SE* = 19), while there was any difference when participants responded with the right hand (Face: 706 ms, *SE* = 16.8; Body: 709 ms, *SE* = 17) (post hoc tests: p < .0004). The ANOVA also revealed a significant Condition x Hand interaction ($F_{(1,23)}$ = 11.549, p = .002): in the Congruent condition right-hand responses (669 ms, *SE* = 16.4) were faster than left-hand responses (706 ms, *SE* = 18.7) (post hoc tests < .0007), while in the Incongruent condition there was any difference in RTs due to response-hand.

Electrophysiological Results

The electrophysiological results of global amplitude analysis, scalp electric field (GFP) analysis and source estimations are reported separately for each comparison (see Supplementary Figs. S1-S7).

FF-I vs. FF-C

The global amplitude analysis (see Supplementary Figs. S1-A; S2) revealed three periods of significant ERP modulation: 1) from 132 to 196 ms after S2 onset, in particular over central clusters of electrodes (right and midline location) from 132 to 168 ms, and over posterior clusters of electrodes, more sustained on a right location, during the whole time period (around P10, $t = -4.22$, $p = .0005$); it is compatible with a N170 modulation, with a typical posterior right topography, indexing the stage of structural encoding during faces processing[13]. It was characterized by higher amplitude in response to Incongruent than to Congruent condition. 2) from 200 to 250 ms after S2 onset, in particular over central clusters of electrodes, at a left and midline location, over left anterior cluster of electrodes, and over posterior clusters of electrodes, at a right and midline location (around C5, $t = 5.34$, $p = .00004$); it is compatible with a fronto-central N200 modulation with higher amplitude to Congruent than to Incongruent condition indexing the recognition of congruent emotions[23] 3) from 418 to-464 ms after S2 onset, in particular over central clusters of electrodes (at a left and midline location) from 418 to 444 ms, and over posterior clusters of electrodes, at a central and right location, during the whole time window (around FC1, $t = -3.16$, $p = .005$); it is compatible with a N400 modulation of higher amplitude to Incongruent than to Congruent condition.

The GFP analysis (see Supplementary Fig. S1-B) showed one period of sustained difference between conditions, from 136 to 160 ms after S2 onset.

BB-I vs. BB-C

The global amplitude analysis (see Supplementary Figs. S1-C; S3) revealed three periods of significant ERP modulation: 1) from 138 to 274 ms after S2 onset, in particular over posterior clusters of electrodes from 166 to 228 ms, over anterior and central clusters of electrodes (in particular at a right location) from 240 to 274 ms after S2 onset, and over anterior and central clusters of electrodes (more sustained at a midline location) from 138 to 240 ms after S2 onset (around C2, $t = 5.01$, $p = .00008$); it is compatible with a fronto-central N200 modulation, of higher amplitude in response to Congruent than to Incongruent condition indexing the recognition of congruent emotions[23] 2) from 370 to 554 ms after S2 onset, in particular over central clusters of electrode from 370 to 440 ms (at a midline and left location), from 464 to 554 ms over anterior clusters of electrodes, and over posterior cluster of electrodes during the whole time period (around CP1, $t = -3.66$, $p = .002$); it is compatible with a N400 component, of higher amplitude for Incongruent than for Congruent condition; 3) from 682 to 706 ms after S2 onset over anterior, central and posterior electrodes at a right location (around FT8, $t = -3.27$, $p = .004$). It is compatible with a posterior-central Late Positivity (LP) of higher amplitude in response to Congruent than to Incongruent condition [25,68].

The GFP analysis (see Supplementary Fig. S1-D) showed five period of sustained difference between conditions: 1) from 128 to 216 ms; 2) from 252 to 326 ms; 3) from 406 to 556 ms; 4) from 898 to 942 ms; 5) from 970 to 1000 ms after S2 onset.

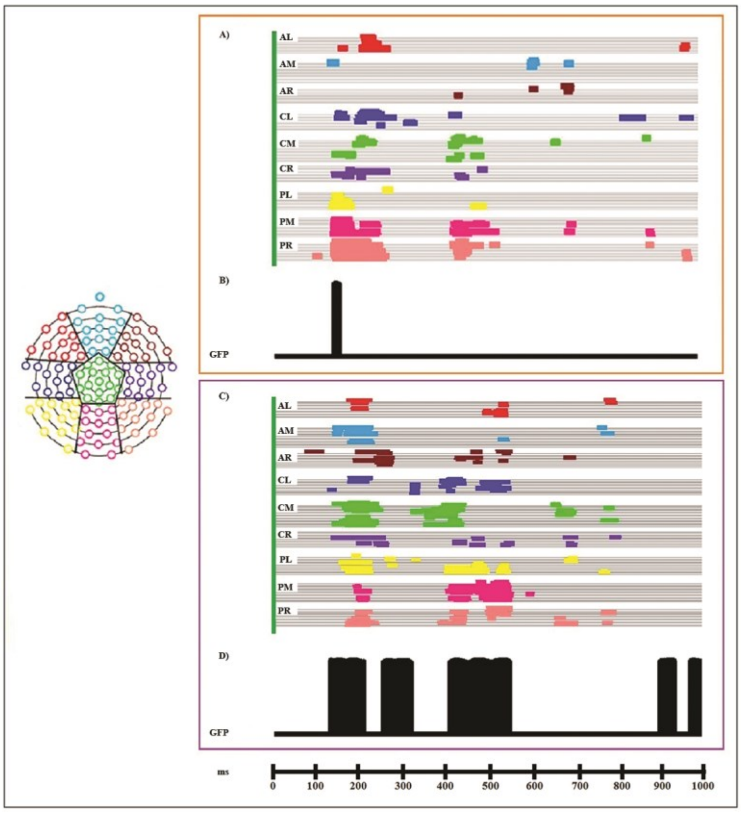

Supplementary Fig.S1

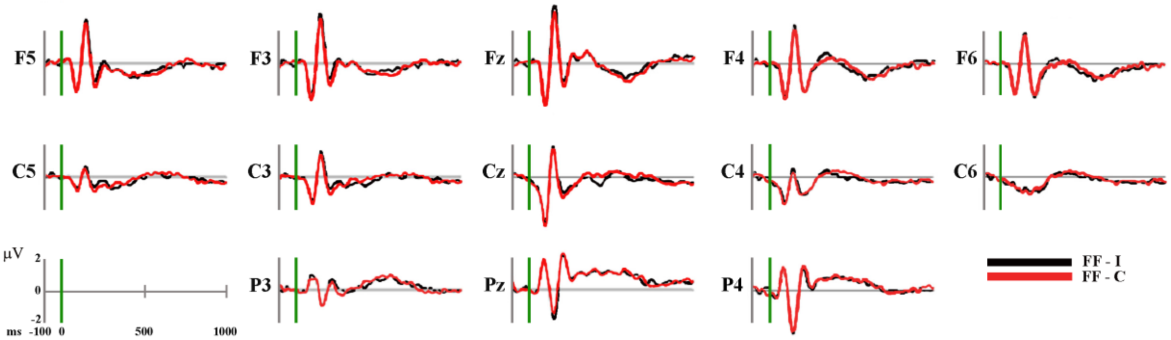

Supplementary Fig. S2

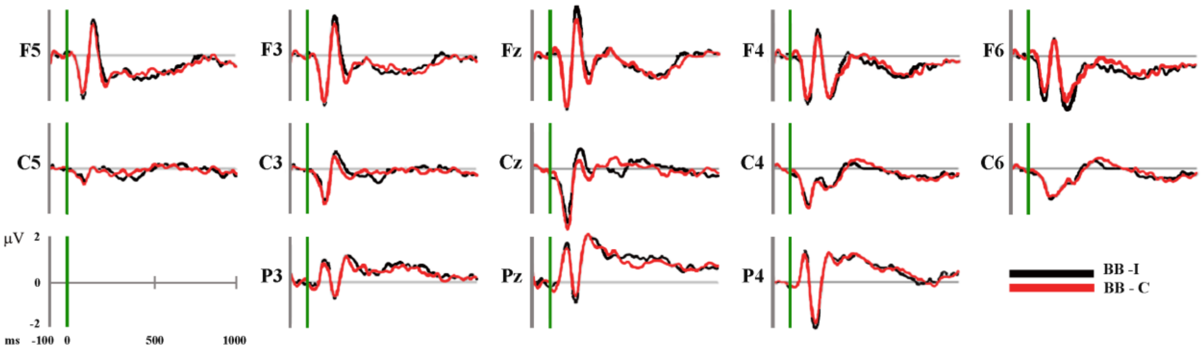

Supplementary Fig. S3

Source estimations

For the first time period of different topography (188-244 ms after S2 onset) significant higher activity in BB-I as compared with BB-C (see Fig. 4B, orange bar; Supplementary Fig. S4-A, orange outline, in red; Supplementary table 1) was found in different brain areas including: bilateral PMc and pre-supplementary motor area (pre-SMA) (BAs 6, 8) extending in ACC (BAs 24, 32) on the right hemisphere. In the same time period, higher activity in BB-C condition (see Fig. S4-A, orange outline, in blue; Supplementary table 1) was found, among others, in left occipital cortex (BAs 17-19) and in bilateral somatosensory-related cortices (BA 5, 7).

In the third period of topographic modulation (676-702 ms after S2 onset) significant higher activation in BB-I was found in (see Fig. 4B, purple bar; Supplementary Fig. S4-A, purple outline, in red; Supplementary table 1) left IFG (BAs 44, 45), while higher activity in BB-C (see Supplementary Fig. S4-A, purple outline, in blue; Supplementary table 1) was found in right MTG (BAs 21, 39) and in medial frontal gyrus (BAs 10, 32).

Supplementary Table 1. Source localization of topographic maps: comparison between BB-I and BB-C condition. Significant results of the statistical comparisons of LAURA source estimations in significant TANOVA time periods are reported with t and p values, Talairach

and Tournoux coordinates (x,y,z) and anatomical labels of solution points with the local maximum different activities.

Condition	TANOVA time period	*t* value	*p* value	Talairach coordinates (x,y,z) mm	Brain region label
**BB-I > BB-C**	**188-244 ms**	3.16	.005	18,-2,24	Right caudate
		2.92	.008	48,-17,18	Right insula, BA[1] 13
		2.83	.01	-63,-19,14	Left inferior temporal gyrus, BA 21
		2.52	.01	-18,0,59	Left middle frontal gyrus, BA6
**BB-C > BB-I**		-4.63	.0001	-33,-76,7	Left middle occipital gyrus, BA 19
		-2.86	.01	-11,-53,34	Left precuneus, BA 31
		-2.67	.01	-26,35,43	Left middle frontal gyrus, BA 8
		-2.47	.02	11,-37,54	Right Paracentral lobule, BA 5
**BB-I > BB-C**	**676-702 ms**	2.67	.01	-56,3,3	Left superior temporal gyrus, BA 22
**BB-C > BB-I**		-3.08	.006	56,-54,6	Right middle temporal gyrus, BA 39
		-2.55	.02	18,48,8	Right medial frontal gyrus, BA 10

BA = Brodmann Area

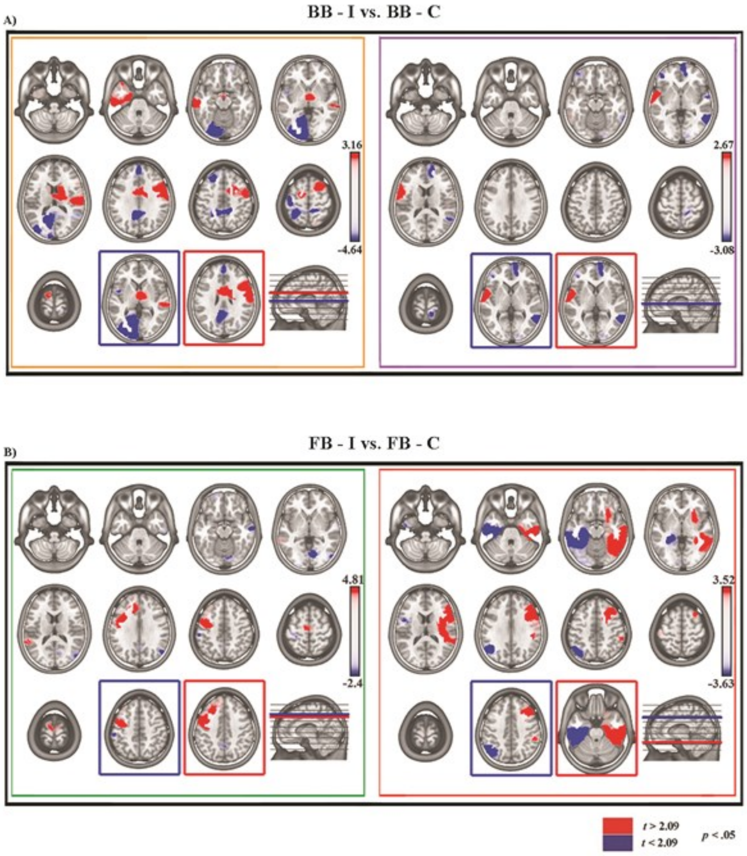

Supplementary Fig. S4

FB-I vs. FB-C

The global amplitude analysis (Supplementary Figs. S5-A; S6) revealed six periods of significant ERP modulation:

1) from 194 ms to 250 ms after S2 onset, in particular over central clusters of electrodes (more sustained at a midline location) from 194 ms to 226 ms, and over posterior clusters of electrodes (again, more sustained at a midline location) for the whole time period (around FC1, $t = 3.39$, $p = .003$); it is compatible with a central N200 modulation, with higher amplitude to Congruent than to Incongruent condition indexing the recognition of congruent emotions[23] 2) from 372 ms to 456 ms after S2 onset over central clusters of electrodes at a right and midline location (around C6, $t = -4.66$, $p = .0002$), compatible with a central P300 modulation of higher amplitude to Congruent than to Incongruent condition[25,29]. 3) from 496 ms to 568 ms after S2 onset over anterior, central and

posterior clusters of electrodes, in particular at a left location from 496 ms to 538 ms, and at a central and right location from 500 ms to 568 ms. 4) from 580 ms to 608 ms after S2 onset over posterior left cluster of electrodes, and over anterior and central clusters of electrodes (at a midline and right location). These two windows (3 and 4) are compatible with an extended N400 modulation, of higher amplitude to Incongruent than to Congruent condition (around P5, $t = -6.44$, $p = .00001$) from 626 ms to 716 ms after S2 onset, starting over posterior cluster of electrodes and then extending over the whole scalp and 6) from 726 ms to 786 ms after S2 onset, in particular over anterior and central cluster of electrodes (at a midline and left location) from 738 ms to 786 ms, and over central and posterior clusters of electrodes (more sustained at a right location) for the whole time period (around P2/O2, $t = -4.94$, $p = .0001$). They are considered as a LP modulation with higher amplitude to Congruent than Incongruent condition[25,68].

The analysis of the GFP (Supplementary Fig. S5-B) showed two period of sustained difference between conditions: 1) from 514 to 540 ms; 2) from 652 to 688 ms after S2 onset.

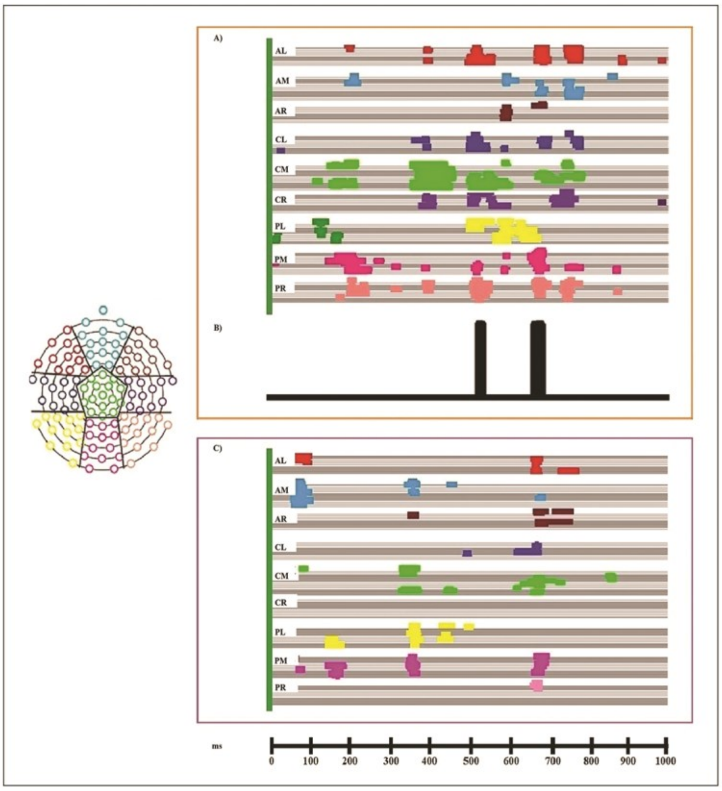

Supplementary Fig. S5

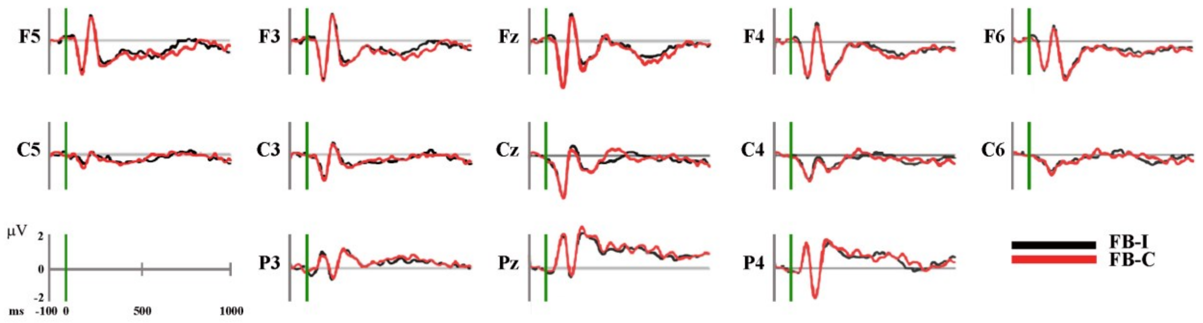

Supplementary Fig. S6

Source estimations

For the first time period of different topography (386-454 ms after S2 onset) significant higher activity in FB-I as compared with FB-C (Fig. 5B, green bar; Supplementary Fig. S4-B, green outline, in red; Supplementary table 2) was found in left PMc extending toward IFG (BAs 6, 9) and left prefrontal cortex encompassing-ACC (BAs 8, 9 32). Higher activity in FB-C (Supplementary Fig. S4-B, green outline, in blue; Supplementary table 2) was found in right occipital cortex (BAs 18, 30).

In the third significant TANOVA period (736-762 ms) higher activity in FB-I (Fig. 5B, red bar; Supplementary Fig. S4-B, red outline, in red; Supplementary table 2) was found in a number of areas, including right occipitotemporal and parahippocampal regions (BAs 19-22, 35, 37, 41), IPL (BA 40), precentral and postcentral gyrus (BAs 2, 6) and IFG (BAs 9, 44, 45). Higher activity in FB-C (Fig. S4-B, red outline, in blue; table 3) was found in left occipitotemporal and parahippocampal regions (BAs 20, 28, 35-37) and in left IPL (BAs 39, 40).

Supplementary Table 2. Source localization of topographic maps: comparison between FB-I and FB-C condition. Significant results of the statistical comparisons of LAURA source estimations in significant TANOVA time periods are reported with t and p values, Talairach and Tournoux coordinates (x,y,z) and anatomical labels of solution points with the local maximum different activities.

Condition	TANOVA time period	t value	p value	Talairach coordinates (x,y,z) mm	Brain region label
**FB-I > FB-C**	**386-454 ms**	4.81	.0001	-41,-9,38	Left precentral gyrus, BA[1] 6
		3.42	.0001	-18,27,29	Left medial frontal gyrus, BA 9
**FB-C > FB-I**		-2.39	.03	11,-70,-6	Right lingual gyrus, BA 18
**FB-I > FB-C**	**736-762 ms**	3.52	.002	56,-19,-21	Right fusiform gyrus, BA 20
		3.20	.004	56,26,16	Right inferior frontal gyrus, BA 45
**FB-C > FB-I**		-3.63	.001	-56,-60,41	Left inferior parietal lobule, BA 40
		-3.52	.02	48,-34,-20	Left fusiform gyrus, BA 20

BA = Brodmann Area

BF-I vs. BF-C

The global amplitude analysis (Supplementary Figs. S5-C; S7) revealed three period of significant ERP modulation: 1) from 54 to 102 ms after S2 onset over anterior clusters of electrodes (at a left and midline location) (around Fz, t = -3.61, p = .002), compatible with an anterior N100 of higher amplitude to Incongruent than to Congruent condition 2) from 332 to 374 ms after S2 onset, in particular over posterior clusters of electrodes (at a left and midline location) from 332 to 344 ms after S2 onset, and over central cluster of electrodes for the whole time period (around Oz, t = -2.89, p = .01), identified as a P300 modulation of higher amplitude to Congruent than to Incongruent condition[25,29] 3) from 656 to 692 ms after S2 onset, in particular over left and right anterior clusters of electrodes from 656 to 676 ms after S2 onset, and over central and posterior cluster of electrodes

(mainly at a midline location) for the whole time period (around PO4, t = -4.01, p = .0007), compatible with a central-posterior LP modulation of higher amplitude to Congruent than to Incongruent condition[25,68].

The GFP analysis did not show periods of sustained difference between conditions.

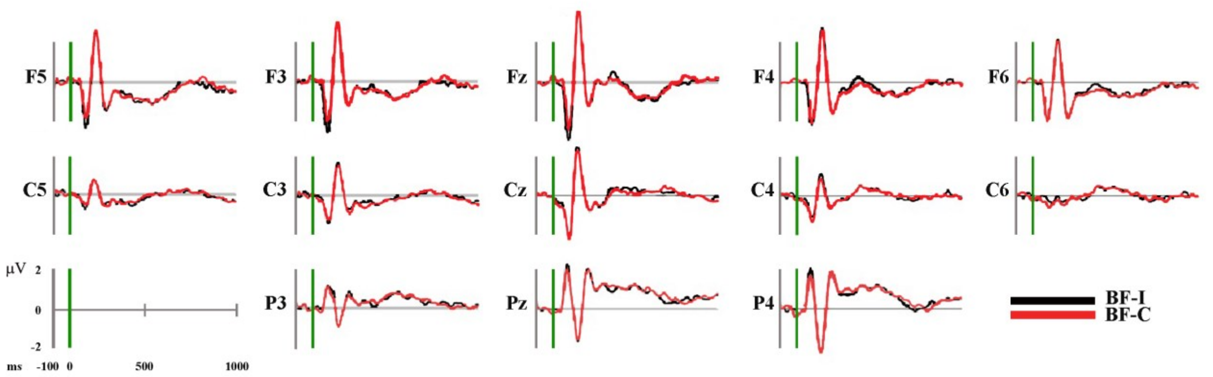

Supplementary Fig. S7

Discussion

BB-I vs BB-C

The global amplitude analysis showed a first period of significant ERP modulation between BB-I and BB-C around 140-270 ms after the S2 onset, compatible with a frontocentral N200 component of higher amplitude in response to BB-C than BB-I condition (between 190-240 ms). Of note, a previous study reported a similar N200 modulation in response to semantically congruent actions[23], likely indexing their recognition. Our results further extend these previous findings, showing that this N200 modulation could also index the recognition of congruent emotions. During this period of amplitude modulation, the TANOVA suggested the involvement of different neural generators in the two conditions.

The source analysis showed in BB-C condition, higher activation, among others, in regions of the left ventral stream related to body processing[4,5] and in bilateral precuneus and PCC, involved in processing of emotion-specific information from different stimulus type[64]. Among activated areas, it is interesting to note the involvement of right parietal regions (Supplementary Fig. S4-A, orange outline). Since the parietal cortex is also part of the MM, it could be involved in the processing of somatosensory information conveyed by images of body postures expressing emotions, contributing to the comprehension of the observed emotional postures.

Among cerebral regions activated in BB-I condition, it is noteworthy the involvement of the right ACC, and of both bilateral pre-SMA and PMc (Supplementary Fig. S4-A, orange outline). The activation of ACC during the affective incongruence between S1 and S2, is in accord with its role in conflict monitoring and/or resolution[65], also in emotional tasks with a cognitively demanding component[66,67].

Regarding bilateral PMc and pre-SMA, our results are consistent with previous evidence that during the observation of emotional body postures there is an activation of these brain areas[8].

Hence, these results suggest that the activation in both conditions of different regions pertaining to the MM (somatosensory-related cortices in the Congruent vs. bilateral premotor regions in the Incongruent condition) could contribute to the comprehension of the emotion conveyed by bodies.

The last significant amplitude modulation between conditions, emerged between 676-702 ms, in a time window corresponding to the typical latency range of the LP, with higher amplitude in response to BB-C than BB-I. Considering the explicit nature of our task, it likely reflected categorization and evaluation processes of the emotional content of congruent postures[25,68]. The TANOVA confirmed the overlapping of significant topographic difference between conditions, and the source analysis revealed the activation of the left IFG in BB-I and of mPFC in BB-C condition (Supplementary Fig. S4-A, purple outline).

FB-I vs FB-C

The global amplitude analysis showed a period of significant ERP modulation between about 370-450 ms after S2 onset, in a time window corresponding to the latency range of the P300 component, with higher amplitude in response to FB-C than FB-I condition, probably indexing the recognition process underpinned by cortical body- and face areas[25]. The TANOVA confirmed the overlapping of significant topographic difference between conditions, and the source analysis established the activation of right occipital regions in FB-C (Supplementary Fig. S4-B, green outline). The activation of left ACC in FB-I condition suggest its higher cognitively demanding component[65-67]. Interestingly, activation of left PMc and IFG emerged during FB-I condition (Supplementary Fig. S4-B, green outline), likely indexing the involvement of MM for action representation and comprehension.

The last significant amplitude modulation between conditions, emerged between 736-762 ms, in a time window corresponding to the latency range of the LP, with higher amplitude in response to FB-C than FB-I, likely reflecting categorization and evaluation processes of the emotional content of congruent postures[25,68], as for BB-C condition. The TANOVA confirmed the overlapping of significant topographic difference between conditions, and the source analysis revealed a common activation of temporal and parahippocampal regions. The significant activation in FB-I condition of right IFG, PMc and ACC could index the higher effort required to solve its double level conflict and to make a correct evaluation.

References

60. Frank, M.G. & Stennett, J. The forced-choice paradigm and the perception of facial expressions of emotion. *J. Pers. Soc. Psychol.* **80,** 75-85 (2001).

61. Tracy, J.L., Robins, R.W. & Schriber, R. A. Development of a FACS-verified set of basic and self-conscious emotion expressions. *Emotion*. **9,** 554-9 (2009).

62. Spinelli, L., Andino, S.G., Lantz, G., Seeck, M. & Michel, C.M. Electromagnetic inverse solutions in anatomically constrained spherical head models. *Brain Topogr*. **13,** 115-25 (2000).

63. Talairach, J. & Tournoux, P. Co-planar stereotaxic atlas of the human brain. New York (NY) (Thieme Medical Publisher, 1988).

64. Kim, J. et al. Abstract representations of associated emotions in the human brain. *J. Neurosci.* **35,** 5655-63 (2015).

65. Botvinick, M.M., Cohen, J.D. & Carter, C.S. Conflict monitoring and anterior cingulate cortex: an update. *Trends Cogn. Sci*. **8,** 539-46 (2004).

66. Kerns, J.G. et al. Anterior cingulate conflict monitoring and adjustments in control. *Science.* **303(5660),** 1023-6 (2004).

67. Müller, V.I. et al. Incongruence effects in crossmodal emotional integration. *Neuroimage.* **54,** 2257-66 (2011).

68. Wu, Y.C., Coulson, S. Meaningful gestures: electrophysiological indices of iconic gesture comprehension. *Psychophysiology*, **42,** 654–67 (2005).

Figure Legends

Fig. S1 Electrophysiological results of "intra-category" session: global ERP amplitude analysis and global electric field strength analysis.

(A) Statistical analysis of global ERP amplitude. Periods of significant differences of ERP amplitude (p < .05; duration ≥ 20 ms) at each electrode and time point between FF-I and FF-C conditions are displayed as colored horizontal lines. Each horizontal line represents one scalp electrode. Different colors indicate different clusters of electrodes; the distribution of the clusters over the electrode montage is shown in the inset on the left side of the figure. AL: anterior left; AM: anterior midline; AR: anterior right. CL: central left; CM: central midline; CR: central right. PL: posterior left; PM: posterior midline; PR: posterior right. (B) Global scalp electric field analyses: statistical analysis of global electric field strength. Black areas indicate time intervals of significant

differences (p < .05; duration ≥ 20 ms) of Global Field Power (GFP) between FF-I and FF-C conditions. (C) Statistical analysis of global ERP amplitude. Periods of significant differences of ERP amplitude (p < .05; duration ≥ 20 ms) at each electrode and time point between BB-I and BB-C conditions are displayed as colored horizontal lines. Each horizontal line represents one scalp electrode. Different colors indicate different clusters of electrodes; the distribution of the clusters over the electrode montage is shown in the inset on the left side of the figure. AL: anterior left; AM: anterior midline; AR: anterior right. CL: central left; CM: central midline; CR: central right. PL: posterior left; PM: posterior midline; PR: posterior right. (D) Global scalp electric field analyses: statistical analysis of global electric field strength. Black areas indicate time intervals of significant differences (p < .05; duration ≥ 20 ms) of Global Field Power (GFP) between BB-I and BB-C conditions.

Fig. S2 ERP waveforms for S2 in the two experimental conditions: FF-I and FF-C at selected electrodes.

Group-averaged (n = 20) S2-locked ERP waveforms, plotted as voltage in μV as function of time in ms (stimulus onset: 0 ms). Black: FF-I; red: FF-C.

Fig. S3 ERP waveforms for S2 in the two experimental conditions: BB-I and BB-C at selected electrodes.

Group-averaged (n = 20) S2-locked ERP waveforms, plotted as voltage in μV as function of time in ms (stimulus onset: 0 ms). Black: BB-I; red: BB-C.

Fig. S4 Statistical comparison of LAURA source estimations between BB-I and BB-C and between FB-I and FB-C over significant TANOVA time intervals.

(A) All significant voxels are colored ($t(19) > 2.09$ / < -2.09, $p < .05$): positive t values (red color) indicate higher current source densities in BB-I than in BB-C; negative t values (blue color) indicate higher current source densities in BB-C than in BB-I. LAURA solutions are rendered on MNI152 template brain (left hemisphere on the left side). Orange outline: first significant TANOVA time interval (188-244 ms after S2 onset). Purple outline: third significant TANOVA time interval (676-702 ms after S2 onset). (B) All significant voxels are colored ($t(19) > 2.09$ / < -2.09, $p < .05$): positive t values (red color) indicate higher current source densities in FB-I than in FB-C; negative t values (blue color) indicate higher current source densities in FB-C than in FB-I. LAURA solutions are rendered on MNI152 template brain (left hemisphere on the left side). Green outline: first significant TANOVA time interval (386-454 ms after S2 onset). Purple outline: third significant TANOVA time interval (736-762 ms after S2 onset).

Fig. S5 Electrophysiological results of "cross-category" session: Global ERP amplitude analysis and global electric field strength analysis.

(A) Statistical analysis of global ERP amplitude. Periods of significant differences of ERP amplitude ($p < .05$; duration $\geq$ 20 ms) at each electrode and time point between FB-I and FB-C conditions are displayed as colored horizontal lines. Each horizontal line represents one scalp electrode. Different colors indicate different clusters of electrodes; the distribution of the clusters over the electrode montage is shown in the inset on the left side of the figure. AL: anterior left; AM: anterior midline; AR: anterior right. CL: central left; CM: central midline; CR: central right. PL: posterior left; PM: posterior midline; PR: posterior right. (B) Global scalp electric field analyses: statistical analysis of global electric field strength. Black areas indicate time intervals of significant

differences (p < .05; duration ≥ 20 ms) of Global Field Power (GFP) between FB-I and FB-C conditions. (C) Statistical analysis of global ERP amplitude. Periods of significant differences of ERP amplitude (p < .05; duration ≥ 20 ms) at each electrode and time point between BF-I and BF-C conditions are displayed as colored horizontal lines. Each horizontal line represents one scalp electrode. Different colors indicate different clusters of electrodes; the distribution of the clusters over the electrode montage is shown in the inset on the left side of the figure. AL: anterior left; AM: anterior midline; AR: anterior right. CL: central left; CM: central midline; CR: central right. PL: posterior left; PM: posterior midline; PR: posterior right.

Fig. S6 ERP waveforms for S2 in the two experimental conditions: FB-I and FB-C at selected electrodes.

Group-averaged (n = 20) S2-locked ERP waveforms, plotted as voltage in μV as function of time in ms (stimulus onset: 0 ms). Black: FB-I; red: FB-C.

Fig. S7 ERP waveforms for S2 in the two experimental conditions: BF-I and BF-C at selected electrodes.

Group-averaged (n = 20) S2-locked ERP waveforms, plotted as voltage in μV as function of time in ms (stimulus onset: 0 ms). Black: BF-I; red: BF-C.
